# Chicken gga-miR-1306-5p targets *Tollip* and plays an important role in host response against *Salmonella enteritidis* infection

**DOI:** 10.1186/s40104-019-0365-2

**Published:** 2019-07-15

**Authors:** Weiwei Sun, Ranran Liu, Peng Li, Qinghe Li, Huanxian Cui, Maiqing Zheng, Jie Wen, Guiping Zhao

**Affiliations:** Institute of Animal Sciences, Chinese Academy of Agricultural Sciences, State Key Laboratory of Animal Nutrition, Beijing, 100193 China

**Keywords:** gga-miR-1306-5p, LPS, Pro-inflammatory cytokine, *Salmonella enteritidis*, *Tollip*

## Abstract

**Background:**

Increasing evidence indicates that microRNAs (miRNAs) are involved in inflammatory response and immune regulation following pathogen invasion. The purpose of this study was to elucidate the roles played by *Gallus gallus* microRNA-1306-5p (gga-miR-1306-5p) in host responses against potential invasion by *Salmonella enteritidis* (SE) in chickens and the underlying mechanisms.

**Results:**

In present study, the expression levels of gga-miR-1306-5p were determined in both tissues and HD11 cells. The results showed that gga-miR-1306-5p was significantly increased following SE infection or lipopolysaccharide (LPS) stimulation. The dual luciferase reporter assay further validated that gga-miR-1306-5p targeted the Toll-interacting protein (*Tollip*), and thereby participated in the regulation of immune response against SE or LPS stimulation through binding with the 3′-untranslated region (3’UTR) of *Tollip*. Additionally, the expression of *Tollip* was significantly blocked by over-expressed gga-miR-1306-5p. The underlying mechanisms by which gga-miR-1306-5p modulated the production of pro-inflammatory cytokines were also investigated. Molecular biological assays demonstrated that overexpression of gga-miR-1306-5p promoted the production of pro-inflammatory mediators, including *NF-κB*, *TNF-α*, *IL-6*, and *IL-1β*, which produced effects similar to those of *Tollip* knockdown.

**Conclusions:**

Taken together, gga-miR-1306-5p induced by SE or LPS, regulates the immune response by inhibiting *Tollip*, which activates the production of inflammatory cytokines. This study has provided the first direct evidence that gga-miR-1306-5p targets *Tollip*, and is involved in the host response against SE.

## Background

*Salmonella enteritidis* (SE) is currently the only *Salmonella* serotype that frequently causes human illness via meat or egg contamination [1]. The on-farm environment of chickens is a rich source of multiple *Salmonella* serotypes, which can pose a severe threat to food safety [2]. Although SE contamination can be effectively controlled through various measures in the poultry industry, the number of reported SE cases continues to increase [3]. Additionally, the resistance of SE to multiple antimicrobial agents has been a great challenge in the treatment of animal and human diseases [4]. Therefore, it is necessary to develop novel preventive and therapeutic strategies for SE infection.

Innate immunity is the first line of host defense barriers against invading infectious pathogens [5]. The recognition of microbial pathogens is an essential step in activation of the host innate immune response, which relies on several kinds of germline-encoded pattern recognition receptors (PRRs) [6]. Toll-like receptors (TLRs) are the most extensively studied PRRs and crucial players required for hosts to eliminate invading microbial pathogens [7, 8]. The Toll interacting protein (*Tollip*), a TLR-associated signaling protein, is an endogenous regulator in the TLR signaling pathway [9]. Increasing evidence suggests that *Tollip* is required to maintain immune homeostasis and control the *MyD88*-dependent *NF-κB* activation pathway during inflammation by suppressing *IRAK-1* activity, confirming a modulatory role of *Tollip* in the immune response [10].

miRNAs are a group of small non-coding RNA molecules (18–25 nucleotides in length), that regulate a variety of biological processes by reducing gene expression at the post-transcriptional level [11]. As reported previously, up to 60% of all protein-coding genes have been predicted to be regulated by miRNAs to a certain extent [12]. Furthermore, miRNAs induce mRNA degradation or repress protein translation mainly through binding to the 3′-untranslated region (3′UTR) of target mRNAs [13]. Compelling evidence based on data analyses and experimental verification reveals that miRNAs not only regulate protein-encoding genes, but are also closely correlated with increasing complexity in multi-cellular organisms [14]. Therefore, these small RNAs have been demonstrated to play critical roles in diverse biological processes, including the cell cycle, proliferation and differentiation, apoptosis, and pathogenesis [15]. Moreover, studies have reported that miRNAs have important functions in regulating the innate immune response induced by bacteria [16, 17]. Presently, an array of miRNAs have been researched in the regulation of TLR pathways at different levels, including regulation of TLR expression, TLR-associated signaling proteins, TLR-induced transcription factors, and the production of functional cytokines [18].

In present study, our data show that *Gallus gallus* microRNA-1306-5p (gga-miR-1306-5p) was increased not only in various tissues following SE infection but also in HD11 cells stimulated by lipopolysaccharide (LPS). A subsequent molecular study confirmed that gga-miR-1306-5p targeted *Tollip*, and the highly expressed gga-miR-1306-5p significantly blocked the expression of *Tollip*. Furthermore, overexpression of gga-miR-1306-5p upregulated the release of pro-inflammatory mediators, including nuclear factor kappaB (*NF-κB*), tumor necrosis factor (*TNF-α*), interleukin (IL)-6, and *IL-1β*, which ultimately produced effects similar to those of *Tollip* silence. Taken together, gga-miR-1306-5p induced by SE or LPS, regulates the immune response by inhibiting *Tollip*, which activates the production of inflammatory cytokines. Our data provide valuable information on the roles of miRNAs during the development of SE infection, as well as new insight into the defense mechanisms of the innate signaling pathway in chickens.

## Methods

### Sampling and challenge

Specific-pathogen-free white leghorn chickens were obtained from the Beijing Laboratory Animal Research Center (Beijing, China) and were raised in climate-controlled, fully enclosed isolation facilities at the experimental center of China Agricultural University (Beijing, China) under identical management conditions. These healthy chickens were randomly divided into two groups for the stimulation experiment. The experimental group was orally challenged with 1 mL PBS containing 10^8^ CFU of SE and the other group was correspondingly challenged with 1 mL PBS as the control. The chickens were respectively killed at 0, 12, 48, and 72 h after treatment, and tissues (spleen, caecum, cecal tonsil, and bursa fabricius) were dissected, snap-frozen and preserved at − 80 °C for later use.

### Cell culture and LPS exposure

HD11 cells were grown at 37 °C and 5% CO_2_ in RPMI-1640 medium containing 10 mmol/L HEPES, 1 mmol/L sodium pyruvate, 1% glutamine, 1% MEM NEAA, 10% fetal bovine serum, and 5% chicken serum (Gibco, USA). HEK293 cells were cultured in DMEM high-glucose medium (HyClone, USA) containing 10% fetal bovine serum under 5% CO_2_ and 37 °C.

For the LPS exposure, we used the LPS (Sigma, USA) from *Salmonella enterica* serotype enteritidis to stimulate HD11 cells *in vitro*. HD11 cells were challenged with 100 ng/mL LPS and harvested at different times (0, 3, 6, 12, and 24 h) for RNA extraction. Non-stimulated HD11 cells were collected as the control, and each experiment had three biological replicates.

### Real-time quantitative PCR for miRNA and mRNA

Total RNA was extracted from tissues or HD11 cells using the MiniBEST Universal RNA Extraction Kit (Takara, Japan). RNA was quantified using a NanoDrop ND-2000 spectrophotometer (NanoDrop Products, USA). cDNA for mRNA was synthesized from 100 μg RNA using a High-Capacity cDNA Reverse Transcription Kit (Thermo Fisher Scientific, USA), and the harvested cDNA was stored at − 20 °C for later use.

Real-time quantitative PCR using SYBR® Premix Ex Taq™ (Takara, Japan) was performed on a QuantStudio™ real-time PCR system (Applied Biosystems, USA). The amplification reaction volume of 12 μL contained 6.0 μL SYBR® Premix Ex Taq™, 1 μL cDNA sample, 0.24 μL ROX Reference Dye, 0.5 μL of each primer (10 mmol/L), and 3.76 μL ddH_2_O. The thermal cycling conditions were 30 s at 95 °C, followed by 40 cycles of 3 s at 95 °C and 34 s at 60 °C. A dissociation curve was conducted after each assay to determine target specificity. Primers of the target genes were designed with Primer Premier 5 software, and β-actin was used as the reference gene [19].

To synthesize the cDNA for the miRNA analysis and quantify mature gga-miR-1306, the specific stem-loop primer for gga-miR-1306 (GTCGTATCCAGTGCGTGTCGTGGAGTCGGCAATTGCACTGGATACGACACTGGAC) and the 5S rRNA reverse primer were incubated together with the total RNA of each sample, and then reverse transcribed with SuperScript® III Reverse Transcriptase (Invitrogen, USA). The relative miRNA expression level was normalized to 5S rRNA expression.

The primer sequences used for mRNA and miRNA quantification are listed in Table 1. And the primers for *TNF-α* were referenced from the excellent work by Rohde et al. [20]. The relative mRNA and miRNA expression levels were calculated using the 2^−ΔΔCt^ method [21]. Three independent replications were used for each assay and data are presented as means ± SEM.

**Table 1:** Primer sequences for qRT-PCR and the plasmid construction

Name	Primer sequence (5′ →3′)	Accession number	Purpose
*gga-miR-1306-5p*	F: gACCACCTCCCCTGCAA	NR-035019	qRT-PCR
	R: CAGTGCGTGTCGTGGAGT		
*Tollip*	F: ATGATCGCATTGCTTGGACA	NM-001006471	qRT-PCR
	R: AAAGACGTGTATGACATCACC		
*NF-κB*	F: TGACCGCCAATAGCTTGTCC	NM-205129	qRT-PCR
	R: ACAGCTAAATGCAATGCCGTTC		
*IL-6*	F: TTCGACGAGGAGAAATGCCT	NM-204628	qRT-PCR
	R: CGACGTTCTGCTTTTCGCTAT		
*IL-1β*	F: TGGGCATCAAGGGCTACAAG	NM-204524	qRT-PCR
	R: CCAGGCGGTAGAAGAAGATGAAG		
*β-action*	F: GAGAAATTGTGCGTGACATCA	NM-205518	qRT-PCR
	R: CCTGAACCTCTCATTGCCA		
*gga-5 s-rRNA*	F: CCATACCACCCTGGAAACGC	NR-046276	qRT-PCR
	R: TACTAACCGAGCCCGACCCT		
*Tollip*	F: TCGCTCGAGGCCACCATGAATCACTTCCTGCGTTGT	NM-001006471	Cloning
	R: CCAGCGGCCGCGATGACCACCCGTTTTAT		

### Plasmid construction

To construct a *Tollip*-3′UTR reporter vector, the full length 3′UTR region of *Tollip* was amplified from cDNA derived from healthy chickens. The PCR products were digested with NotI and XhoI, which were then cloned into the psiCHECK™-2 luciferase reporter vector (Promega, USA). The mutant of *Tollip*-3′UTR was constructed using the Fast Site-Directed Mutagenesis Kit (Tiangen, China) with specific primers. The putative binding site (TTGC … … GAGGTTGG) for gga-miR-1306-5p was mutated into TTGC … … TATGTTTG. All recombinant plasmids were extracted with the Endotoxin-Free Plasmid DNA Miniprep Kit (Tiangen, China) and confirmed by Sanger sequencing before the dual-luciferase reporter assay.

### miRNA mimics and inhibitor

The gga-miR-1306-5p mimics (double-stranded chemically modified RNA oligonucleotides) and inhibitor (single-stranded chemically modified RNA oligonucleotides) obtained from Ribobio (Guangzhou, China) were respectively utilized to over-express or inhibit miRNA activity in cells, respectively. Negative control mimics or negative control inhibitors were transfected as the respective controls. The gga-miR-1306-5p mimic sequences were 5′-ACCACCUCCCCUGCAAACGUCCAGU-3′ (sense), 5′-UGGACGUUUGCAGGGGAGGUGGUUU-3′ (antisense); negative control mimic sequences were 5′-UUCUUCGAACGUGUCACGUTT-3′ (sense), 5′-ACGUGACACGUUCGGAGAATT-3′ (antisense); gga-miR-1306-5p inhibitor sequence was 5′-ACUGGACGUUUGCAGGGGAGGUGGU-3′ and the negative control inhibitor sequence was 5′-CAGUACUUUUGUGUAGUACAA-3′. Transfection for each kind of oligonucleotide in HD11 cells was performed with the *Trans*IT-TKO® reagent (Mirus Bio, USA) in accordance with the manufacturer’s instructions. HD11 cells were seeded into 12-well plates and incubated overnight. The cells were subsequently transfected with 100 nmol/L of each kind of oligonucleotide for 36 h, and then stimulated with LPS for another 3 h.

### Target mRNA prediction

To further explore the underlying molecular mechanisms of gga-miR-1306-5p regulating the innate immune response, its downstream targets were predicted by the bioinformatics databases, including Findtar (http://bio.sz.tsinghua.edu.cn) and RNAhybrid (http://bibiserv.techfak.uni-bielefeld.de/rnahybrid/submission.html).

### Transient transfection and dual-luciferase reporter assays

To verify the hypothesis that gga-miR-1306-5p targets *Tollip*, we performed the cotransfections and dual-luciferase reporter assays. Specifically, HEK293 cells were seeded and cultured in 12-well plates. A 100 nmol/L concentration of the miRNA mimics, or controls were co-transfected with the wild-type or mutated *Tollip*-3′UTR luciferase reporter vector into HEK293 cells to identify the miRNA target. We determined the concentrations of plasmids by Nanodrop 2000 spectrophotometer (Thermo Scientific, USA), and the amount of wild-type or mutant-type *Tollip*-3′UTR plasmid was about 3 μg. Transient transfections were performed using Lipofectamine 3000™ (Invitrogen, USA) according to the manufacturer’s protocol. The cells were collected after 36 h for the dual-luciferase reporter assay system (Promega, USA) according to the manufacturer’s instructions and the relative luciferase activity value was achieved against the *Renilla* luciferase control. The results were obtained from three independent experiments performed in triplicate.

### RNA interference

The *Tollip*-specific small interfering RNA (siRNA) sequences were 5′-GGAACAAGAAUGCAGCUAUTT-3′ (sense) and 5′-AUAGCUGCAUUCUUGUUCCTT-3′ (antisense). The *Tollip*-specific siRNA transfection was performed with *Trans*IT-TKO® reagent (Mirus Bio, USA) in accordance with the manufacturer’s instructions. HD11 cells were first seeded into 12-well plates and incubated overnight. The cells were subsequently transfected with 100 nmol/L siRNA against *Tollip* or control siRNA for 24 h, and then stimulated with LPS for another 3 h.

### Western blotting

After each transfection, HD11 cells were collected and lysed with the RIPA buffer. Proteins were then extracted from the cells. Whereafter, SDS-PAGE was performed with equal amounts of protein, after which the proteins were transferred onto polyvinylidene difluoride membranes in a semidry manner. The membranes were blocked with 5% non-fat milk at room temperature for 1 h, and then incubated with primary antibodies against *Tollip* (Abcam, UK) or β-actin (Abcam, UK) for 1 h at room temperature. Subsequently, the membranes were incubated with the secondary antibody conjugated with HRP at room temperature for 1 h. At last, the immunoreactive proteins were detected using the Tanon 5200 instrument, and digital imaging was performed with a cold CCD camera.

### Statistical analysis

All data are presented as mean ± SEM, and Student’s *t*-test was applied to compare the difference of means between two groups in the GraphPad Prism 7 software. A *P*-value < 0.05 was considered as statistically significant.

## Results

### SE infection significantly promotes gga-miR-1306-5p expression

The expression levels of gga-miR-1306-5p in four SE infected chicken tissues, including the spleen, bursa fabricius, cecal tonsil, and cecum were measured.

**Fig. 1:**
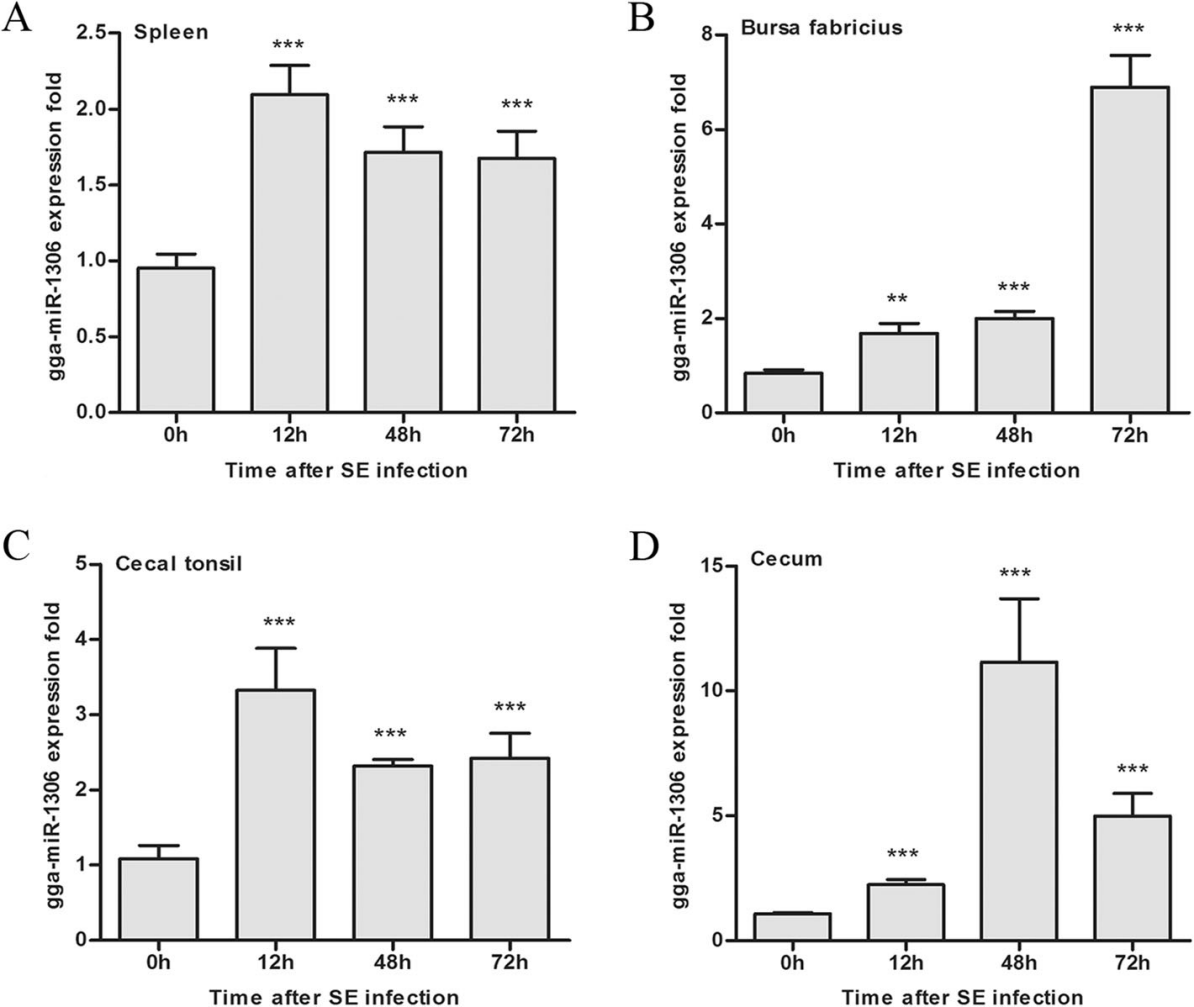
The expression profiles of gga-miR-1306-5p detected by qPCR relative to 5S rRNA. The time-course expression profiles of miR-1306-5p in the spleen (**a**), bursa fabricius (**b**), cecal tonsil (**c**), and cecum (**d**) after SE infection at 0, 12, 48, and 72 h are shown. ^*^P<0.05, ^**^P<0.01, ^***^P<0.001

The results showed that after infection by SE, the expression levels of gga-miR-1306-5p in all these four tissues were significantly increased (Fig. 1). Specifically, gga-miR-1306-5p was rapidly promoted and reached a peak at 12 h after SE infection in the spleen and cecal tonsil samples. The expression of gga-miR-1306-5p in the cecum was significantly increased and reached a peak at 48 h post-infection. Consistently, the expression profile of gga-miR-1306-5p in bursa fabricius samples was also determined, showing significant time-dependent upregulation.

### gga-miR-1306-5p facilitates LPS-induced inflammatory cytokine production

**Fig. 2:**
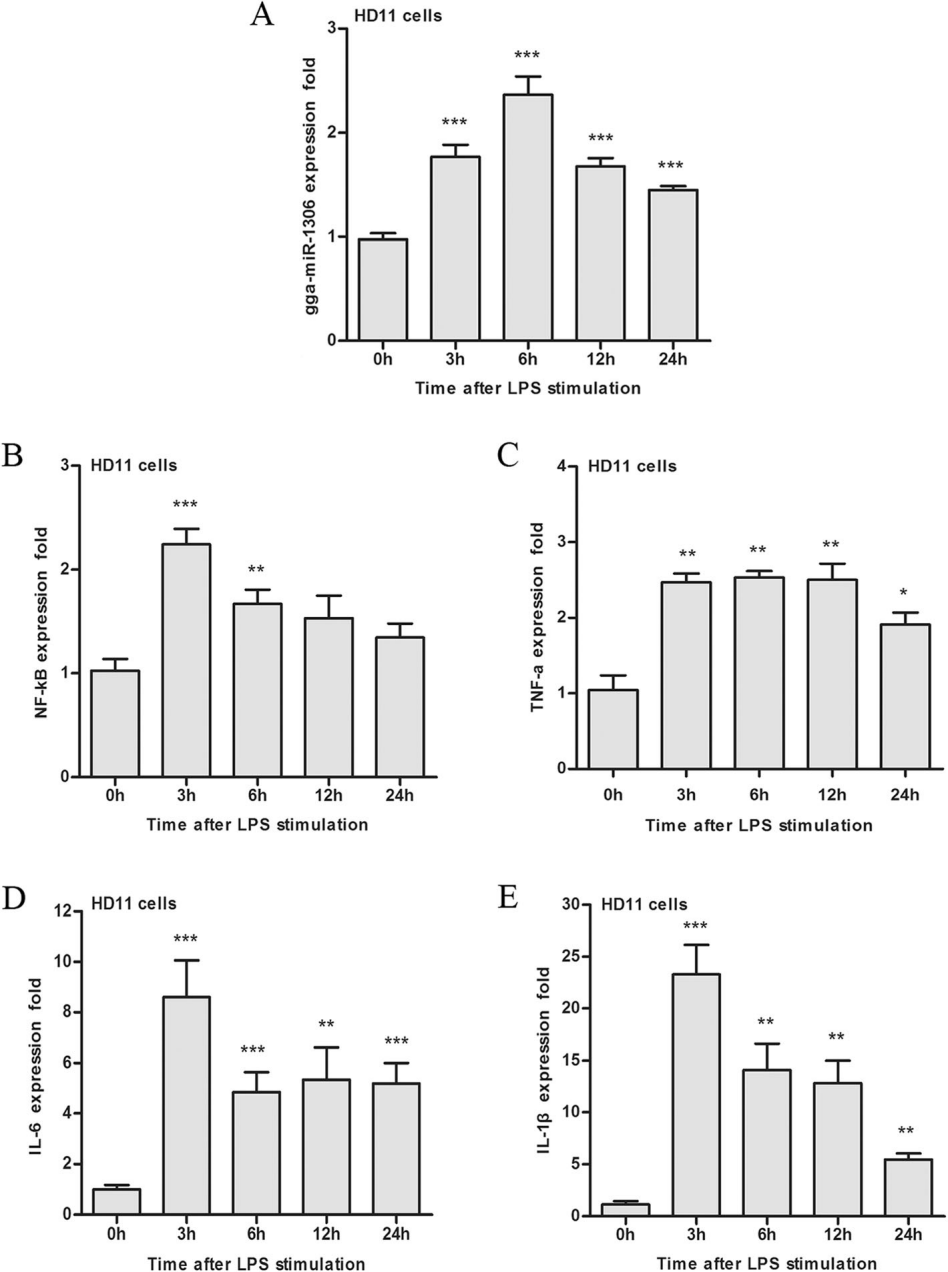
The mRNA expression profiles of gga-miR-1306-5p (**a**), and downstream pro-inflammatory mediators including *NF-κB* (**b**), *TNF-α* (**c**), *IL-6* (**d**), and *IL-1β* (**e**) after LPS stimulation in HD11 cells are given. The data are expressed as the mean ± SEM from three independent experiments performed in triplicate. ^*^P<0.05, ^**^P<0.01, ^***^P<0.001

As shown in Fig. 2a, LPS stimulated HD11 cells and activated gga-miR-1306-5p expression, which was significantly up-regulated over time, with gga-miR-1306-5p reaching a peak value at 6 h post-infection.

Additionally, the expression profiles of four pro-inflammatory mediators including *NF-κB*, *TNF-α*, *IL-6*, and *IL-1β* *in vitro* were measured. As shown in Fig. 2b-e, the pro-inflammatory cytokines were rapidly induced and subsequently released after LPS stimulation in HD11 cells. The expression levels of *NF-κB*, *TNF-α*, *IL-6*, and *IL-1β* were all significantly up-regulated in HD11 cells, reaching the peak values of release after being stimulated at 3 h.

### *Tollip* is the target of gga-miR-1306-5p

**Fig. 3:**
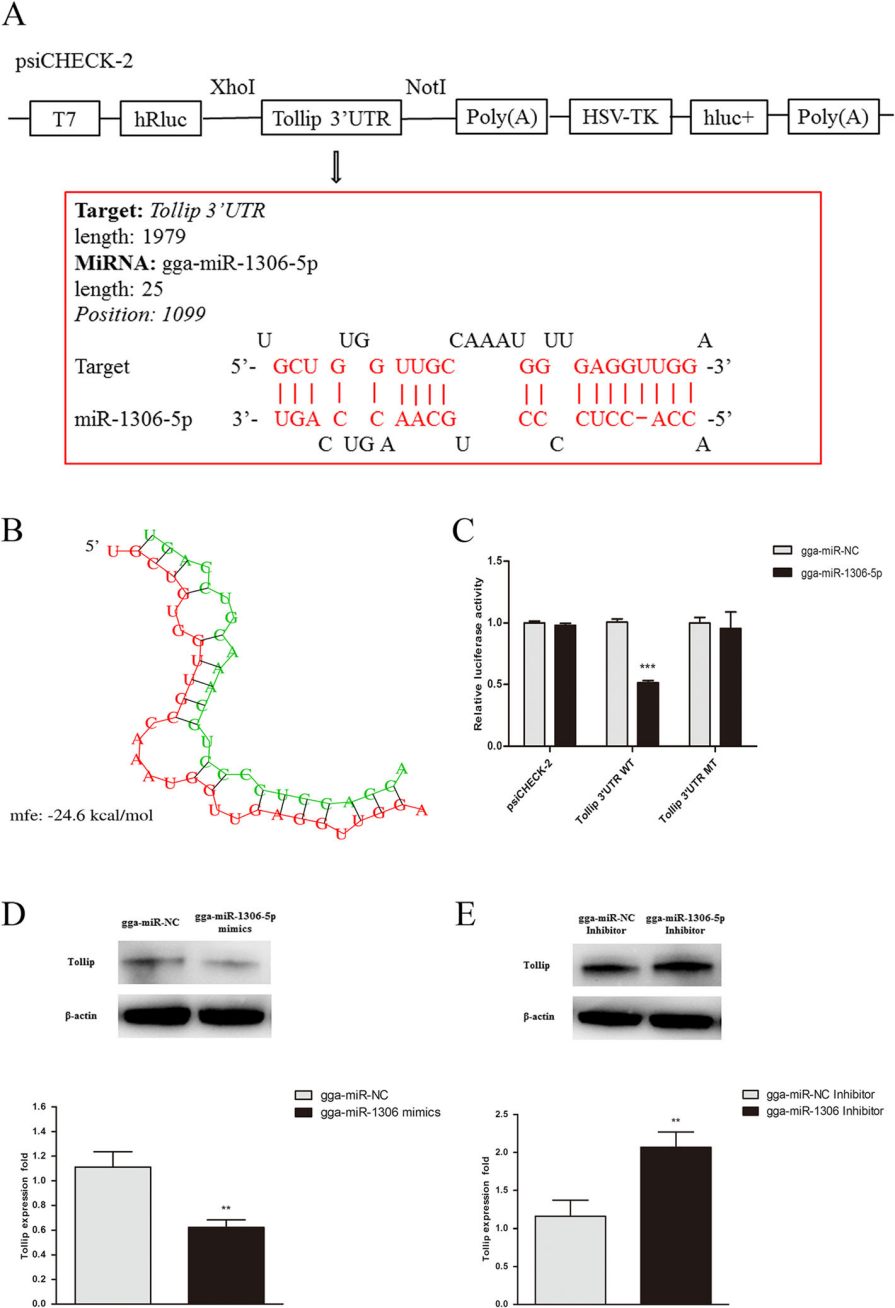
*Tollip* is a target of gga-miR-1306-5p. **a** The sequence alignment of miR-1306-5p and one target site in the 3’UTR of *Tollip* are shown. **b** The illustration demonstrates the binding between the target of *Tollip* 3’UTR and gga-miR-1306-5p. **c** HEK293 cells were cotransfected with 3 μg of psiCHECK-2 or *Tollip* 3’UTR luciferase reporter plasmid (WT or MT), along with control mimics or gga-miR-1306-5p mimics as indicated. Firefly luciferase activity was measured and normalized by Renilla luciferase activity to finally calculate the Relative luciferase activity, after 36 h post transfection. **d** Effects of gga-miR-1306-5p mimics on *Tollip* expression at both protein and mRNA levels compared with negative control. **e** Effects of gga-miR-1306-5p inhibitor on *Tollip* expression at both protein and mRNA levels compared with control inhibitor. ^*^P<0.05, ^**^P<0.01, ^***^P<0.001

The preliminary prediction indicated that *Tollip* was a candidate target of gga-miR-1306-5p, and the potential binding site in the 3′UTR of *Tollip* was found for gga-miR-1306-5p (Fig. 3a). A more intuitive diagram demonstrated the binding between the target of *Tollip* 3′UTR and gga-miR-1306-5p (Fig. 3b).

Additionally, we performed the dual-luciferase reporter assays on HEK293 cells 36 h post-cotransfection to validate the binding of gga-miR-1306-5p with the candidate target *Tollip*. As shown in Fig. 3c, the gga-miR-1306-5p mimics down-regulated the luciferase activity compared with the control mimics when cotransfected with the *Tollip*-3′UTR reporter plasmid in HEK293 cells. Nevertheless, the inhibition of luciferase activity was attenuated after cotransfection with the mutant-type vector of *Tollip* 3′UTR and gga-miR-1306-5p. Collectively, the luciferase reporter assays demonstrate that gga-miR-1306-5p may be the regulator of *Tollip* in chickens.

To confirm the functions of gga-miR-1306-5p in the regulation of *Tollip*, we explored the protein and mRNA expression levels of *Tollip* in HD11 cells treated with the mimics and inhibitor of gga-miR-1306-5p. As shown in Fig. 3d and e, overexpression of gga-miR-1306-5p suppressed *Tollip* expression at the protein and mRNA levels, whereas the suppression of *Tollip* was restrained in the presence of the gga-miR-1306-5p inhibitor. Combined with the results of the luciferase reporter assays, these data collectively reveal that gga-miR-1306-5p targ**e**ts the 3′UTR of *Tollip*.

### gga-miR-1306-5p regulates *Tollip* and downstream inflammatory mediator expression

**Fig. 4:**
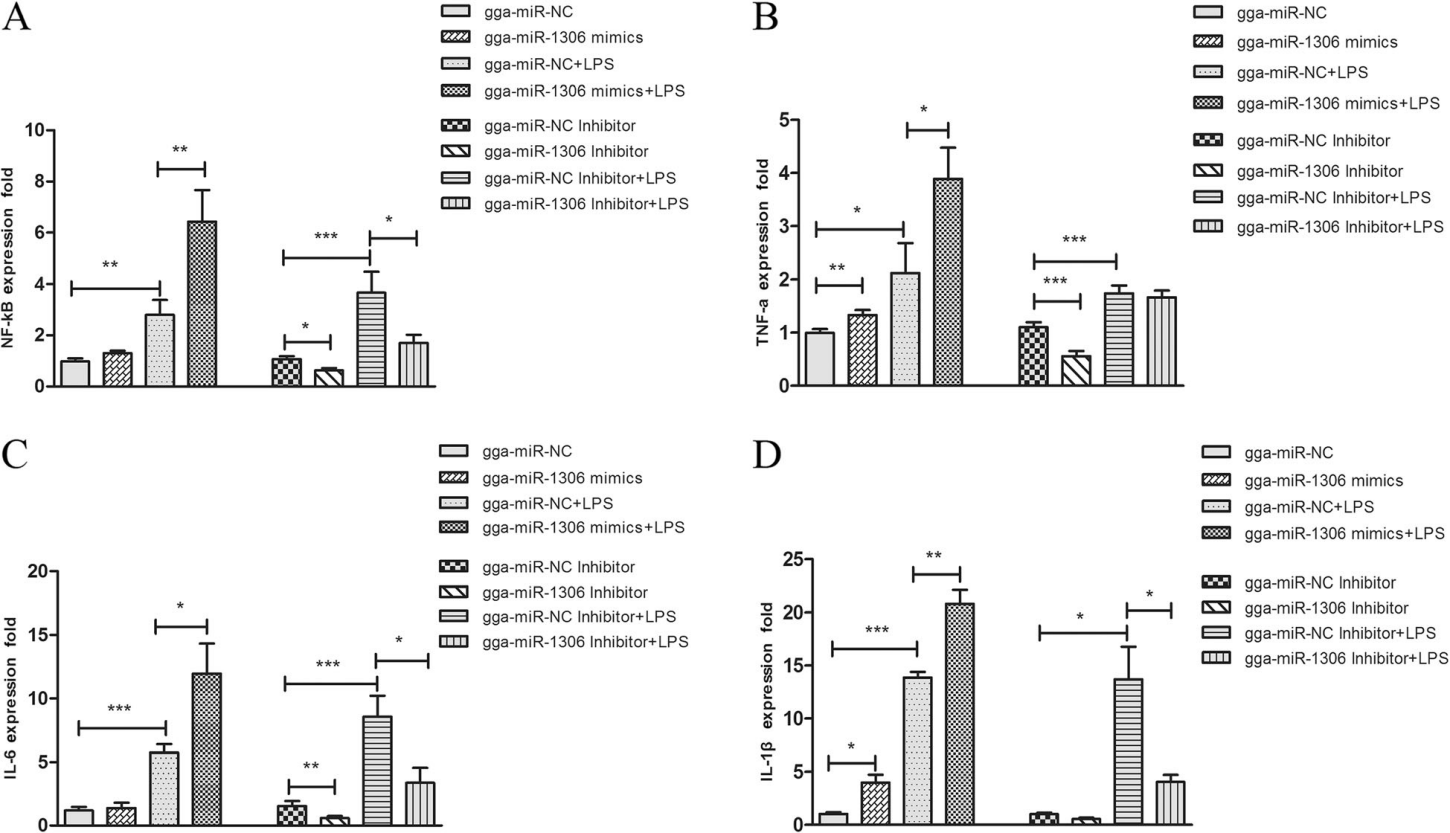
The HD11 cells were transfected with gga-miR-1306-5p mimics or negative control and gga-miR-1306-5p inhibitor or control inhibitor within a final concentration of 100 nmol/L. At 36 h posttransfection, HD11 cells were stimulated with LPS and after another 3 h, the expression levels of four pro-inflammatory mediators including *NF-κB* (**a**), *TNF-α* (**b**), *IL-6* (**c**), and *IL-1β* (**d**) were then analyzed by qPCR. The data are expressed as the mean ± SEM from three independent experiments performed in triplicate. ^*^P<0.05, ^**^P<0.01, ^***^P<0.001

To evaluate the effects of gga-miR-1306-5p on the bacteria-induced inflammatory response, the mimics, negative control, inhibitor, or control inhibitor were respectively transfected into HD11 cells. And then 36 h post-transfection, the expression levels of four pro-inflammatory mediators were assessed (Fig. 4). The data showed that LPS stimulation promoted the significant upregulation of cytokines and induced the release of inflammatory mediators in HD11 cells. The gga-miR-1306-5p mimics further promoted the release of inflammatory cytokines after transfection compared to the negative control. In contrast, decreases in the *NF-κB*, *TNF-α*, *IL-6*, and *IL-1β* expression were observed following the introduction of gga-miR-1306-5p inhibitor. The same trends were observed after LPS stimulation. Taken together, these results indicate that gga-miR-1306-5p acts as a positive regulator of the innate immune response in chickens.

### *Tollip* siRNA increases the production of inflammatory cytokines

**Fig. 5:**
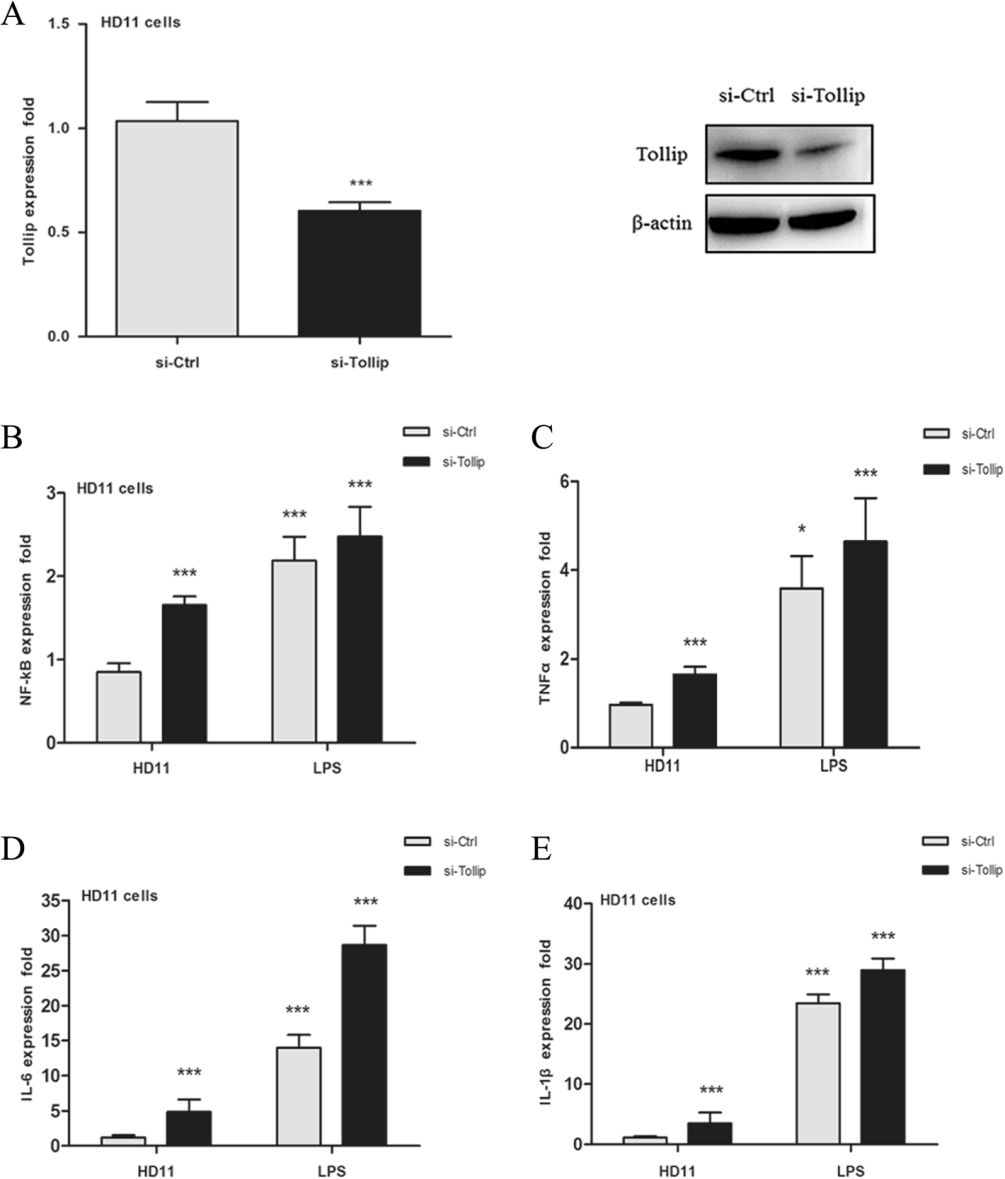
The expression levels of *Tollip* and inflammatory cytokines after *Tollip* interference. **a** The mRNA and protein levels of *Tollip* in HD11 cells transfected with control siRNA (si-Ctrl) or siRNA against *Tollip* (si-*Tollip*). The mRNA expression of four downstream pro-inflammatory mediators including *NF-κB* (**b**), *TNF-α* (**c**), *IL-6* (**d**), and *IL-1β* (**e**) in HD11 cells, which were transfected with si-Ctrl or si-*Tollip* for 24 h, and then stimulated with LPS for another 3 h. The data are expressed as the mean ± SEM from three independent experiments performed in triplicate. ^*^P<0.05, ^**^P<0.01, ^***^P<0.001

To confirm the function of *Tollip* in the regulation of pro-inflammatory cytokines, HD11 cells were transfected with *Tollip*-specific small interfering RNA (siRNA). The results demonstrated that siRNA efficiently suppressed *Tollip* expression at both mRNA and protein levels (Fig. 5a). We silenced *Tollip* and detected the expression profiles of downstream pro-inflammatory factors in HD11 cells stimulated with LPS. As shown in Fig. 5b-e, knockdown of *Tollip* significantly promoted the release of downstream inflammatory mediators, including *NF-κB*, *TNF-α*, *IL-6*, and *IL-1β*. Further stimulation using LPS exacerbated the inflammatory response, which was consistent with the effects of gga-miR-1306-5p overexpression.

**Fig. 6:**
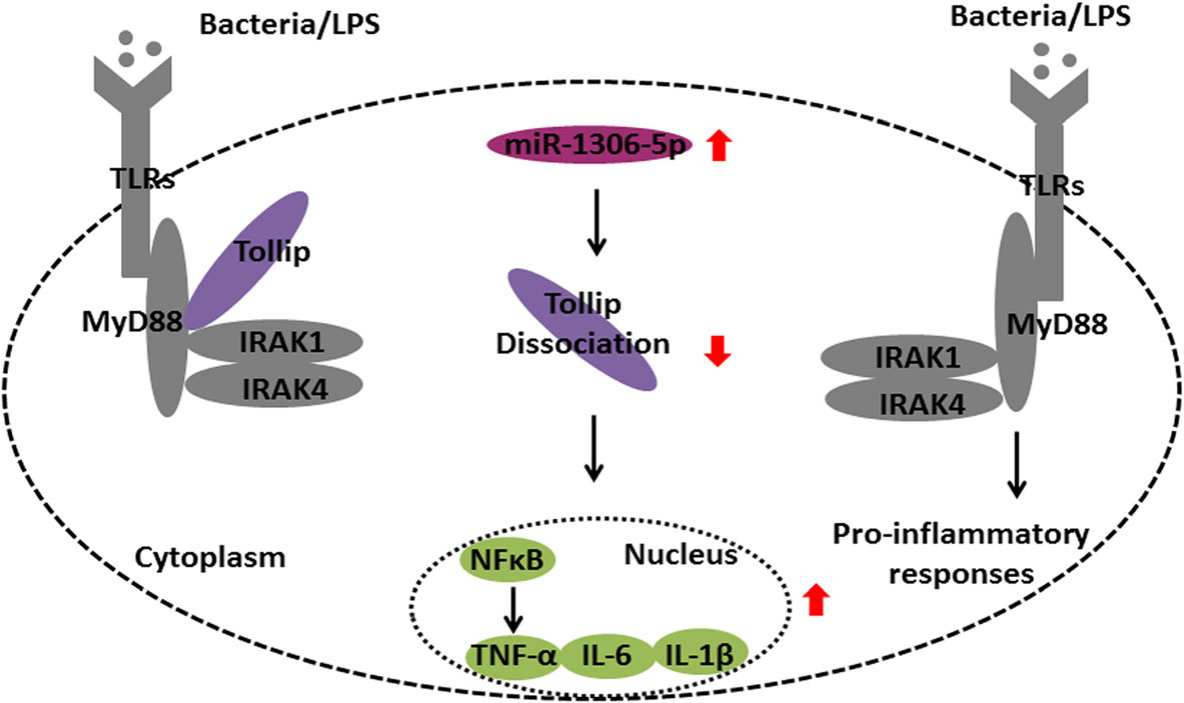
A proposed model of gga- miR-1306-5p regulation on activation of pro-inflammatory responses via targeting *Tollip* in chickens (Red arrow: up-regulation or down-regulation). *Tollip* is required to maintain immune homeostasis and control the *MyD88*-dependent TLR signaling pathway during inflammation. Following pathogen or LPS stimulation, *Tollip* firstly binded with *IRAK-1*, and this *Tollip*-*IRAK-1* complex interacted with *IRAK-4*. The results generated from our study showed gga-miR-1306-5p were significantly increased following SE infection or LPS stimulation. The highly expressed gga-miR-1306-5p could significantly suppress the target *Tollip*, and subsequently facilitate the downstream pro-inflammatory mediators including *NF-κB*, *TNF-α*, *IL-6* and *IL-1β*

As illustrated in Fig. 6, the results of our study collectively demonstrate that the upregulation of gga-miR-1306-5p induced by pathogen infection or LPS stimulation participated in the immune response by inhibiting *Tollip*, and subsequently promoting the production of inflammatory factors.

## Discussion

Growing evidence demonstrates that miRNAs are involved in the regulation of innate and adaptive immunity responses to various bacterial infections, particularly the Gram-negative enteric pathogen SE [22]. The specific roles of diverse miRNAs in response to SE infection have been explored in chickens. Several novel miRNAs, including gga-miR-125b-5p, gga-miR-34a-5p, gga-miR-1416-5p, and gga-miR-1662 play an important role in SE infection by regulating their target genes in the cecum of laying chickens [23]. Several studies have revealed that miR-155 acts as a component of the inflammatory response and plays important roles in the development and activation of immune-related cells [24, 25]. Despite these studies, there is limited information about the function of miRNAs with regard to the host responses following SE infection in chickens. Thus, more investigations are needed to identify the specific roles and functions of miRNAs in regulating the inflammatory responses against SE infection in chickens, with the aim of exploring pathogenesis, protecting animal welfare, reducing economic losses in the poultry industry and keeping food safe.

To date, no study has reported on the specific regulation of immune responses by miRNA-1306-5p, particularly during SE infection in chickens. In other words, the specific functions of miR-1306-5p in the regulation of innate immunity during infection are an undiscovered and verifiable topic that warrants further investigation. Our preliminary deep sequencing data revealed that 32 miRNAs, including gga-miR-1306-5p, exhibited significantly different expression among healthy and SE-infected chickens [26]. Accordingly, we speculate that gga-miR-1306-5p was involved in the regulation of host immune response against SE infection. Moreover, this could be a novel finding on the mechanisms of gga-miR-1306-5p regulating the inflammatory response in chickens. Above all, the expression and functional mechanisms of one miRNA mainly rely on the regulation of the targeted gene in the pathway [27]. Consequently, identifying the target mRNA of each miRNA is critical to thoroughly understand the biological functions of miRNAs due to the nature of their post-transcriptional regulatory effects.

The spleen is the largest immune organ in the body, playing a major role in an immediate innate reaction after recognizing pathogens through filtering antigens from the blood [28]. Bursa fabricius is an immune organ unique to birds, and the cecum tonsil is the largest intestinal-related lymphoid tissue in poultry. In addition, the cecum is the invading pathway for SE infection. Therefore, in the present study, we first detected gga-miR-1306-5p expression levels in these four tissues to identify the roles played by gga-miR-1306-5p. Subsequently, the expression levels of gga-miR-1306-5p were determined in HD11 cells *in vitro*. Our results showed that gga-miR-1306-5p were increased in both various tissues following SE infection and in HD11 cells stimulated by LPS. Thus, the underlying mechanisms by which gga-miR-1306-5p modulated the production of pro-inflammatory cytokines were further investigated. Subsequently, a molecular study confirmed that the nucleotide sequence in the 3′UTR of *Tollip* is the potential target site of gga-miR-1306-5p. As previously mentioned, the expression and functional mechanisms of one miRNA mainly rely on regulation of the targeted gene in the pathway [29]. We provide a more intuitive demonstration in Fig. 6. In the current study, the expression of *Tollip* was significantly inhibited by highly expressed gga-miR-1306-5p. Interestingly, overexpression of gga-miR-1306-5p upregulated the release of pro-inflammatory cytokines, which produced effects similar to that of *Tollip* silence. Collectively, gga-miR-1306-5p, induced by SE or LPS, regulates immune responses by inhibiting *Tollip*, which activates the production of inflammatory cytokines. According to previous reports, *Tollip* limits the production of pro-inflammatory mediators during inflammation and infection [30], and not only has been implicated as a negative regulator of the *NF-κB* signaling pathway but also controls the magnitude of inflammatory cytokine production in response to LPS [10]. Consistent with these studies, our data more definitely confirm that *Tollip* silence significantly promotes the release of downstream inflammatory cytokines, acting as a negative regulatory mediator. One innovative point of our study is addressing the precise molecular mechanisms by which *Tollip* modulates cytokine production.

In the long term, in-depth investigations are necessary to discover the novel targets of miR-1306-5p during SE and other types of bacterial infections. Additionally, our data provide us some hints, more thorough studies are indispensable to develop new techniques, such as a vaccine to specifically modulate the expression of one specific miRNA associated with pathogen infection, which will provide new methods to prevent or even treat bacterial infections with a promising application prospect and huge economic value for practical production.

## Conclusions

The results of this study preliminarily confirmed that upregulation of miR-1306-5p in tissues infected by SE or in HD11 cells stimulated with LPS, promote pro-inflammatory cytokine release by suppressing the target *Tollip*, which is conducive to deepen the understanding of the pathogenesis of SE infection in chickens. In summary, our study provides new insight into understanding the mechanisms of the host immune response to *Salmonella* infection through miRNA-induced systems, and provides guidance on potential vaccine targets.

## Data Availability

All data generated or analyzed during this study are included in this published article.

## Abbreviations

3’UTR: 3′-untranslated region
gga-miR-1306-5p: *Gallus gallus* microRNA-1306-5p
IL: Interleukin
LPS: Lipopolysaccharide
miRNA: MicroRNA
*NF-κB*: Nuclear factor kappaB
PRR: Pattern recognition receptor
SE: *Salmonella enteritidis*
siRNA: Small interfering RNA
TLR: Toll-like receptor
*TNF-α*: Tumor necrosis factor
*Tollip*: Toll-interacting protein
